# Conformable microneedle pH sensors via the integration of two different siloxane polymers for mapping peripheral artery disease

**DOI:** 10.1126/sciadv.abi6290

**Published:** 2021-11-26

**Authors:** Wonryung Lee, Seung-hwan Jeong, Young-Woo Lim, Hyunhwan Lee, Joohyuk Kang, Hyunjae Lee, Injun Lee, Hyung-Seop Han, Shingo Kobayashi, Masaru Tanaka, Byeong-Soo Bae

**Affiliations:** 1 Wearable Platform Materials Technology Center, Department of Materials Science and Engineering, Korea Advanced Institute of Science and Technology (KAIST), Daejeon 34141, Republic of Korea.; 2 Center for Biomaterials, Biomedical Research Institute, Korea Institute of Science and Technology, Seoul 136-791, Republic of Korea.; 3 Graduate School of Medical Science and Engineering, Korea Advanced Institute of Science and Technology (KAIST), Daejeon 34141, Republic of Korea.; 4 Department of Urology, Seoul National University Hospital, Seoul 03080, Republic of Korea.; 5 Samsung Electronics Semiconductor R&D Center, Hawseong 18448, Republic of Korea.; 6 Institute for Materials Chemistry and Engineering, Kyushu University, CE41 744 Motooka, Nishi-ku, Fukuoka 819-0395, Japan.

## Abstract

Flexible microneedles are important tools that allow access to the inside of biological tissue from the outside without surgery. However, it had been hard to realize microneedle sensor arrays on flexible substrates because of the difficulty of attaining a needle with a high Young’s modulus for a selected area on a thin or soft substrate. In this work, we developed a microneedle sensor on a hybrid substrate based on high Young’s modulus epoxy siloxane for the microneedles and low Young’s modulus polydimethylsiloxane for the conformable substrate. Polyaniline was deposited on the microneedle for pH sensing. The mechanical durability of the device was assessed by insertion into pig skin 1000 times. Last, the flexible microneedle pH sensors showed their utility for monitoring pH distribution in rats in a peripheral artery diseases model.

## Teaser

A conformable microneedle biochemical detection platform that can diagnose metabolic disease has been developed.

## INTRODUCTION

Peripheral artery disease (PAD) in a lower limb is the third most prevalent atherosclerotic vascular disease, following coronary artery disease and stroke. It is associated with up to three-fold increased cardiovascular risk and can lead to amputation of a limb in severe cases (*1*). Disrupted blood flow by PAD induces ischemic symptoms such as intermittent claudication and exertional pain (*2*). However, most patients with mild or chronic PAD do not present clinical symptoms to make them aware of disease occurrence or progression (*3*). PAD can be diagnosed and monitored with clinical questionnaires and comparisons of systolic blood pressure between ankle and arm, which is called the ankle-brachial index (ABI) (*4*). Although ABI provides high sensitivity and specificity, it does not allow for evaluation of tissue damage caused by compromised blood flow and hypoxic insult. Monitoring pH changes can adequately reflect the tissue damage caused by ischemic insults because hypoxia promotes anaerobic glycolysis leading to lactic acidosis (*5*). However, pH recording in skin has the shortcoming of inaccuracy due to contaminants such as sweat, water, and cosmetics. Furthermore, normal flora of the skin changes the skin pH level and infectious skin conditions can hamper accurate measurements reflecting vascular perfusion (*6*). For an accurate evaluation of PAD diseases, it is important to have a direct measure of dermal acidity over broad areas. Thus, for mapping the pH distribution of PAD diseases, there are two specifications for diagnostic tools: the ability to penetrate skin and the ability to conform over large areas of skin.

The microneedle platform is one candidate for satisfactory penetration. The earliest microneedles realized on silicon chips (called Utah arrays) penetrated the brain to obtain bioelectrical signals from deep sites for surgery (*7*, *8*). Furthermore, microneedles have been used on outer sensing platforms without surgery by accessing dermal sites through the skin of mammals to measure physiological signals with high precision (*9*). They also measured biochemical parameters including pH (*10*, *11*), glucose level (*12*, *13*), and other diagnostic markers (*14*, *15*). To obtain a large area of coverage and spatial information, flexible electronic systems with high sensitivity have been developed on submicron-thick substrates. To get penetrability and large area coverage simultaneously, a flexible microneedle was developed using polymer to deliver drugs to the skin (*16*–*19*) by accessing the transdermal area without surgery. In addition, electrode or polymer coating on a microneedle can be used to make a sensing application. Thus, such flexible microneedle systems show potential as diagnostic tools able to access dermal sites and cover large areas.

Many challenges still exist to realizing diagnostic tools made of flexible microneedles. First, to provide skin penetrability and conformability to skin simultaneously, the integrated substrate for this device must offer both a high Young’s modulus microneedle (10^2^ to 10^3^ μm) and a submicron-thick conformable substrate (~10 μm) (*20*, *21*). A submicron-thick substrate can make high mechanical stress by wrinkling or crumpling, which causes damage to the interface between needle and substrate. To reduce the mechanical stress between the microneedles and thin substrate, the substrate can be made of low Young’s modulus materials to increase its thickness along with high conformability (*22*, *23*). From the perspective of adhesion, a material with high surface energy is desirable (*24*, *25*); however, low Young’s modulus materials, such as polydimethylsiloxane (PDMS), exhibit low surface energy. Thus, there has been no durability evaluation for insertion into skin of integrated flexible microneedles with high conformability. Next, the microneedle requires high resilience to have long-term mechanical stability. In general, as the Young’s modulus of a material increases, the material shows high cycling reliability due to elastic recovery (*26*). Therefore, to make a desirably conformable microneedle device, it is important to hybridize a low Young’s modulus conformable substrate and high Young’s modulus microneedles with outstanding time-dependent resilience.

In the work reported here, we developed a novel multimicroneedle pH sensor array on soft substrates by integrating two siloxane-based polymers, each with a different Young’s modulus. PDMS and epoxy siloxane were used as the soft substrate and microneedles, respectively. These materials have Young’s moduli of 5 MPa and 3 GPa, respectively. The device could have two advantages through these integrated substrates, including skin penetrability of high Young’s modulus epoxy siloxane and conformability to the skin of low Young’s modulus PDMS, respectively. In addition, epoxy siloxane showed high time-dependent resilience compared with other microneedle materials, including Su-8 (*27*) and silicon (*28*, *29*). A 30-nm-thick Au electrode was deposited on a conformable microneedle hybrid substrate. Parylene was used as the passivation layer. Polyaniline (PANI) was chemically deposited on the Au electrode for pH sensing. All pH sensors were covered with poly(3-methoxypropyl acrylate) (PMC3A), which shows low adhesion to protein. The mechanical stability of the newly developed conformable microneedle pH sensor was assessed by repeated insertion into the skin of a pig. Last, the medical applicability of these conformable microneedle pH sensors was demonstrated by measuring the pH distribution on the dermal layer of a peripheral vascular disease rat model.

## RESULTS AND DISCUSSION

**Fig. 1.**
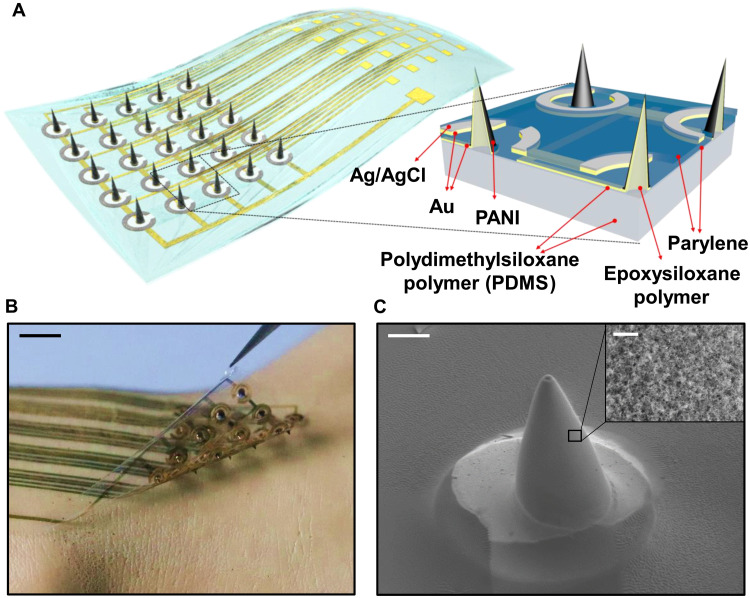
Flexible pH sensor array with epoxy siloxane polymer microneedles. (**A**) Structural schematic of flexible pH sensor array with microneedles. Inset: Cross section of device. (**B**) 5 × 5 pH sensor array with microneedles. Scale bar, 5 mm. Photo credit: Wonryung Lee, Korea Institute of Science and Technology. (**C**) SEM image of a microneedle. Scale bar, 250 μm. Inset: Image of polyamine deposited on a microneedle. Scale bar, 5 μm.

Multipoint microneedle pH sensor arrays were fabricated on 15-μm-thick PDMS (Fig. 1A). The microneedles were made with epoxy siloxane polymer sandwiched between 15-μm-thick PDMS substrate and a passivation layer after plasma treatment. This resulted in highly stable bonding due to a dangling effect and covalent bonds. The chemical structure of the epoxy siloxane polymer is shown in fig. S1. Epoxy siloxane is expected to have excellent biocompatibility because it consists of siloxane bonds (*30*, *31*) with hardness that can be used for microneedles. To prove this, we conducted several in vivo inflammatory response tests and found that the biocompatibility was good enough (fig. S2). A cross section of the conformable microneedle pH sensor array is shown in Fig. 1A (inset). With this integration, the device offers two advantages, flexibility for lamination on round and complicated biosurfaces and penetration ability that allows it to access deep biological sites (Fig. 1B). After gold electrode deposition using a shadow mask process, parylene was deposited as a passivation layer. The sensing window was exposed by reactive ion etching. To measure the pH value, PANI and Ag/AgCl were electrochemically deposited on the open window of the microneedle, which is widely used to fabricate biochemical sensors (*32*–*35*). Figure 1C shows a scanning electron microscope (SEM) image of the microneedle and a magnified view of the PANI surface of the microneedle. The details of the fabrication process and method are shown in fig. S3.

**Fig. 2.**
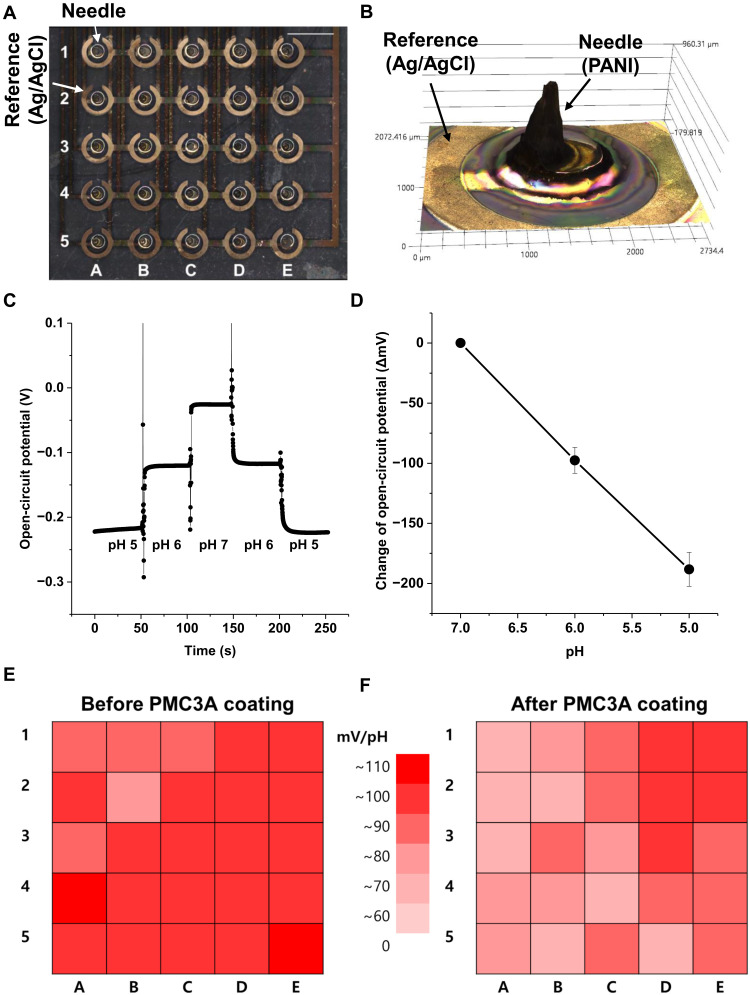
Electrical characteristics of a pH sensor array with microneedles. (**A**) Microscope image of 5 × 5 pH sensor array. Scale bar, 5 mm. Photo credit: Wonryung Lee, Korea Institute of Science and Technology. (**B**) 3D microscope image of a microneedle. (**C**) Open-circuit potential (OCP) time curve of single pH sensor with pH change. (**D**) Change of open circuit potential of 25 pH sensors. (**E**) Distribution of pH sensitivity in 5 × 5 pH sensor array. (**F**) Distribution of pH sensitivity in 5 × 5 pH sensor array after PMC3A coating.

A 5 × 5 microneedle pH sensor array was fabricated to demonstrate its large-area sensitivity and high yield (Fig. 2A). The height (thickness) of the microneedle (900 μm) was measured using a three-dimensional (3D) microscope (Keyence) (Fig. 2B). The sensitivity of the pH sensors was verified by measuring the open circuit potential with phosphate-buffered saline (PBS) of differing pH, which was previously measured using a conventional pH measurement device (Fig. 2C). The average sensitivity of 25 samples in the 5 × 5 pH sensor array was as high as 94 mV/pH (Fig. 2D), which matches well with previously reported values (*16*). The porous structure of PANI causes this high value of pH sensitivity since it is deposited electrochemically (*36*, *37*). Also, the pH sensitivity in a broader range of pH 3 to 7 was demonstrated (fig. S4). Figure 2E shows the distribution of sensitivity in the array. The microneedle device is considered favorable not only in terms of biocompatibility but also in terms of blood compatibility. This matters because there are many chances for microbleeding during injections. Thus, to prevent device degradation caused by microbleeding, the entire device was dip-coated in PMC3A, which is a blood-compatible material (*38*). Evaluation of the blood compatibility was conducted using a control and a PMC3A-coated microneedle. The method of this evaluation is explained in detail in the experimental section. As shown in fig. S5, the microneedle coated with PMC3A has lower platelet activation than the control after the blood compatibility test. Figure 2F shows the distribution of the sensitivity of the pH sensors after coating them with PMC3A. The sensitivity was 82 mV/pH, which was only 13% different from that of the control device. The blood compatibility of dip-coated PMC3A on parylene lasts at least 5 hours in previous reports (*38*). It can be recoated on devices after cleaning by ethanol for reusing to prevent contamination. Therefore, we investigated the comparison of pH sensitivity with primary and secondary PMC3A coatings after the sensor surface was washed with ethanol to remove residues (fig. S6). The data show the only minor difference below 5% after recoating of PMC3A.

**Fig. 3.**
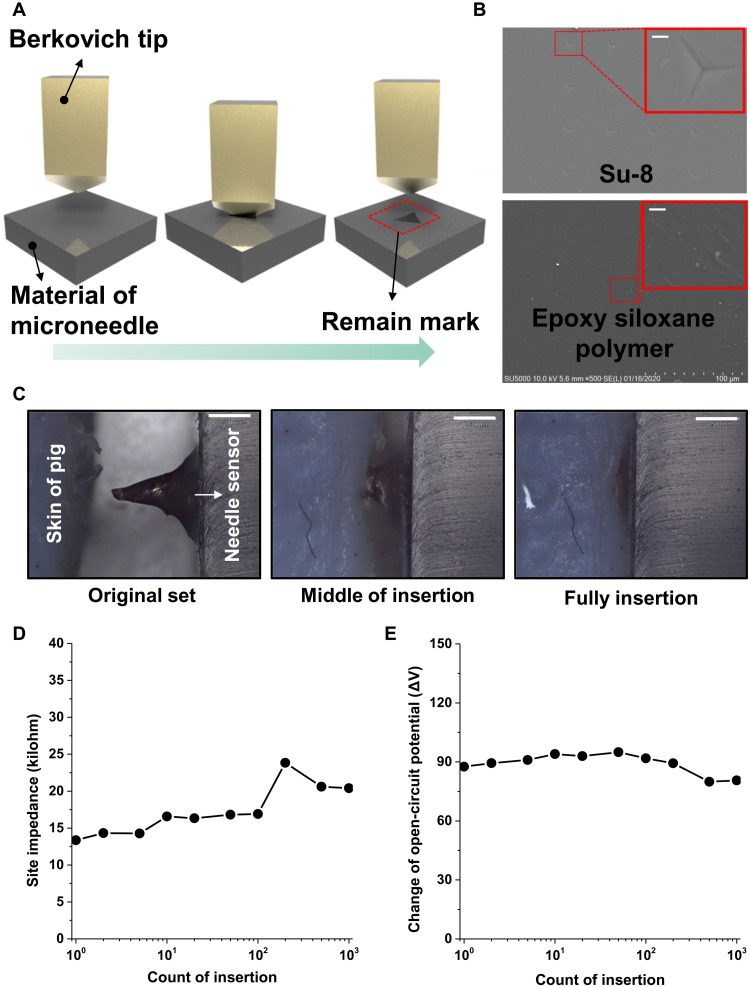
Mechanical characteristics of a pH sensor array with microneedles. (**A**) Schematic of nanoindentation test performance. (**B**) SEM image substrates of Su-8, epoxy siloxane polymer after nanoindentation evaluation. Scale bars, 25 μm (inset) and 5 μm. (**C**) Microscope image of 5 × 5 pH sensor array during cycles of insertion into pig skin. Scale bars, 500 μm. (**D**) Long-term stability of site impedance between PANI electrode and Ag/AgCl reference electrode. (**E**) Long-term stability of OCP curve of a single pH sensor.

Mechanical stability and durability are important factors to ensure multiple uses of the sensors. An important factor to note about these sensors is the brittleness of the microneedles. In general, hardness and elastic compliance are known to be mutually exclusive. However, the microneedles in this sensing platform system should have both the advantages mentioned above, both easy insertion and multiple uses. At first, we performed nanoindentation experiments based on the Oliver-Pharr method to show the hardness and elastic compliance of the epoxy siloxane polymer used in the microneedles (Fig. 3A). The Young’s modulus of the epoxy siloxane polymer was 3 GPa, as calculated by nanoindentation load (*P*)–displacement (*h*) curves (fig. S7). This value is good enough for the needles to penetrate mammal skin, of which humans have skin with a Young’s modulus of only 0.08 to 1.32 MPa (*39*). The important thing is that, in terms of elastic compliance, the epoxy siloxane polymer shows performance superior to those of other high Young’s modulus candidate materials, including silicon and Su-8. Figure 3B shows a SEM image of the samples after the nanoindentation experiment. As can be seen in the figure, there was no remaining indentation mark in the epoxy siloxane polymer after the nanoindentation experiment, whereas silicon and Su-8, which have been used as hard materials for microneedle sensors (*26*), showed remaining marks (figs. S8 and S9).

Furthermore, to demonstrate the durability of the microneedle pH sensors against insertion into the skin, the sensitivity was measured during cycles of insertion into the skin of a pig. Figure 3C shows a microscope image of the microneedle sensors during the insertion cycles. To evaluate the effect on the surface of the microneedles, the site impedance between the electrode coated with PANI on the microneedles and the reference electrode coated with Ag/AgCl was measured (fig. S10). After 1000 insertions into the skin of the pig, there was only a small change of 6 kilohms (Fig. 3D), which was mainly induced by the fat layer of the skin at the site of the electrode. The sensitivity of the pH sensors was also evaluated (Fig. 3E), confirming that there was only 10% degradation after 1000 insertion cycles. In addition to evaluating the microneedle insertion, the mechanical bending stability due to the flexibility of the device was also checked. The bending strain was applied to the flexible microneedle pH sensor using steel bars of various radii. The bend radii were 6, 4.5, 3, and 1.5 mm. As a result, despite being subjected to strain by bend radii of 6, 4.5, 3, and 1.5 mm, sensitivity was degraded by only 17% (fig. S11).

**Fig. 4.**
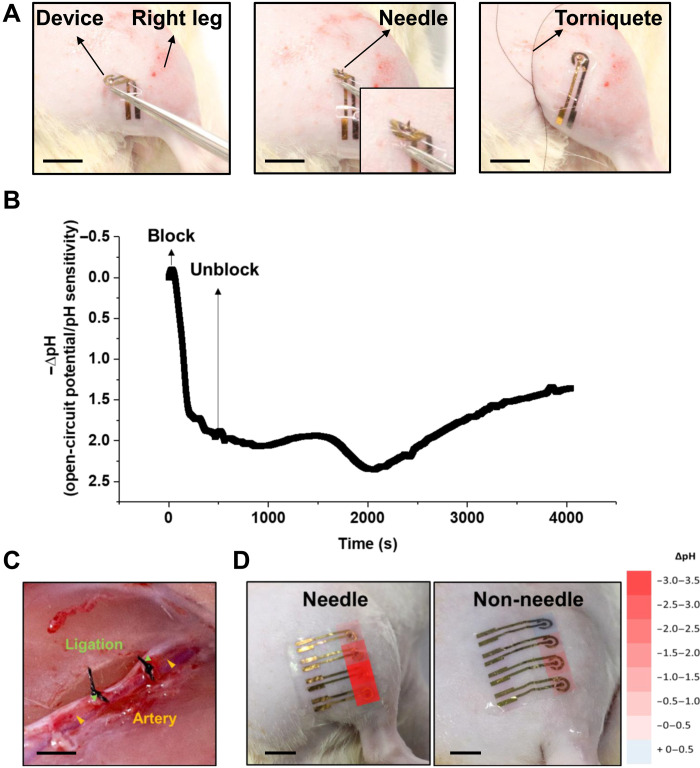
In vivo recording on leg of rat with vascular blockage model. (**A**) Photo of setting of single flexible pH sensor on rat with vascular blockage. Scale bars, 10 mm. (**B**) Recorded change of open circuit potential of a pH sensor with microneedles. (**C**) Photograph of the operation performed with the Peripheral artery disease (PAD) model. Scale bar, 2.5 mm. (**D**) Photograph showing location and pH distribution with microneedle type sensors (left) and non–needle-type sensors (right), respectively. Scale bars, 10 mm. Photo credit: Wonryung Lee, Korea Institute of Science and Technology.

To validate the biomedical applicability of the conformable microneedle pH sensors, we used them to record the dermal pH in a rat model of PAD. The ability to obtain the long-term measurements of our device was demonstrated by applying them on the thigh of an anesthetized rat and measuring the pH profile for 5 hours (fig. S12). Two types of PAD animal models were prepared. One model included physically applying pressure using a tourniquet from outside the skin and the other model included preparation by ligating the artery selectively. First, our newly developed device was applied to the ischemic limb model of a rat. A tourniquet was fastened at the hip joint to block blood flow below the thigh, and the device was placed on the thigh to monitor pH changes, as shown in Fig. 4A. When the tourniquet was fastened appropriately, the pH dropped immediately and showed a plateau at ∆pH value of −1.75 after 5 min. Then, the tourniquet was released to allow blood flow below the thigh and the pH was continuously monitored. The acidity rose for 34 min and then gradually decreased, as shown in Fig. 4B. Data from long-term measurement were filtered using the smooth tools in Origin 9 (fig. S13). The pH increase after tourniquet release reflects reperfusion injury, which is caused by oxidative cell damage following the abrupt restoration of blood flow (*40*).

To mimic PAD in the rat model, the femoral artery was selectively ligated and the pH change of the affected lower limb was measured 1 week after the surgery, as shown in Fig. 4C. To our surprise, the conformable microneedle pH sensor showed gradual pH change of the lower limb from the thigh to the perineal area in the PAD model, while the control model exhibited constant pH distribution over the measured area (fig. S14). The acidity was higher in the distal region than in the proximal region, indicating that damage to the ischemic tissue is more severe with increasing distal distance from the obstructive lesion, as shown in Fig. 4D. Although the non–needle-type conformable pH sensor also reflected the acidity change in the PAD rat, the traditional device was not as sensitive as the needle-type device. Thus, our sensor can be seen to be universally applicable to the technique of biochemical mapping analysis for wearable devices under the skin.

The conformable microneedle pH sensor provided sensitive and durable results on dermal pH changes in the PAD model. The pH change of the manifested lesion represents tissue damages implying disease progression requiring further interventions precedently. Several diseases and conditions—including PAD and diabetes, lymphadenopathy, frostbite, burn, and skin graft monitoring—require evaluation for tissue damages (*41*, *42*). Our conformable microneedle pH sensor is suitable to be used in these situations to provide reliable data and expected to facilitate timely advanced treatments in each patients.

## MATERIALS AND METHODS

### Synthesis of epoxy siloxane

The substances 3-(2-trimethoxysilylethyl)cyclohexene oxide (ShinEtsu), diphenylsilanediol (Sigma-Aldrich), and barium hydroxide monohydrate (Sigma-Aldrich) were mixed in a flask at a molar ratio of 1:1:0.001. The mixed solution was vigorously stirred at 80°C for 4 hours via sol-gel reaction. After the reaction, optically transparent viscous resin was obtained (i.e., epoxy siloxane). Triarylsulfonium hexafluoroantimonate salts (Sigma-Aldrich) were added to the epoxy siloxane at 2 weight % for ultraviolet (UV)–initiated polymerization. Then, the epoxy siloxane was photo-polymerized by UV irradiation at the wavelength of 340 nm. After 1 J of UV irradiation, the epoxy-ring-opening polymerization was completed, and the epoxy siloxane polymer was obtained.

### Evaluation of the mechanical stability

For the evaluation of mechanical stability, a nanoindentation test was conducted using iMicro equipment (KLA). To perform the nano-indentation test, first, epoxy siloxane polymer was spin-coated onto a Si wafer to a thickness of 50 μm. For comparison, 50-μm-thick SU-8 (SU-8 2000, MicroChem) coated on a Si wafer was used. The maximum testing load was 50 mN, and the test was conducted more than 16 times for each sample to ensure accuracy. Representative data from each sample are displayed in the fig. S3. After the test, the surfaces of the epoxy siloxane polymer, SU-8, and the Si wafer itself were specifically inspected using a 3D optical-confocal microscope (VK-X1050, Keyence) and an SEM (SU-5000, Hitachi) to estimate the depth profile.

### Fabrication of a conformable microneedle pH sensor array

PVA [10% (w/v)] was spin-coated at 1000 rpm on glass as a sacrificial layer. PDMS was spin-coated at 2000 rpm as a conformable substrate of the device. The epoxy siloxane was poured on the microneedle mold and vacuumed to remove the air bubble between the epoxy siloxane and the mold. After plasma treatment of the PDMS, the substrate of the device was attached to the microneedle mold. Then, UV light with a shadow mask through the glass was used to polymerize the epoxy siloxane. Last, on the resulting material, PDMS was spin-coated at 3000 rpm. The entire device was annealed at 100°C. On the conformable microneedle substrate, a 50-nm-thick Au electrode was deposited using the polyimide shadow mask. After adding a 1-μm-thick parylene coating for passivation, the sensing electrode was exposed using a reactive ion etcher. Last, PANI and Ag/AgCl were electrochemically deposited on the sensing electrode and measured using an electrochemical analyzer (*16*).

### Recording the pH

The pH-dependent change in the zeta potential of the PANi was measured using an electrokinetic analyzer (SurPASS, Anton Paar). The pH sensor was used under ambient air conditions with real-time open-circuit potential (OCP) measurements from the electrochemical analyzer. The two-electrode method, with PANi as working electrode and a solid-state Ag/AgCl electrode as the counter electrode, was used for the measurement. The pH sensor was calibrated using different pH PBS solutions, with values measured using a conventional pH meter (Eutech).

### Blood compatibility evaluation

PMC3A was dissolved in methanol, and the polymer solution was prepared at 0.2% (w/v). The PMC3A-coated microneedle was prepared by the dip-coating method in that solution and then dried. Human platelet adhesion tests were performed according to a previously reported procedure (*43*). Platelet-rich plasma (PRP) and platelet-poor plasma (PPP) were obtained from human whole blood using two-step centrifugation. A plasma solution containing platelets (4 × 10^7^ cells/cm^2^) was then prepared by mixing the PRP and PPP solutions. The plasma solution (200 μl) was placed on each microneedle substrate, and each substrate was incubated for 1 hour at 37°C. After 1 hour, each substrate was rinsed with PBS, and then the platelets that had adhered to the microneedle substrates were fixed by immersion in 1% glutaraldehyde in PBS for 120 min at 37°C. Last, each microneedle substrate was rinsed with PBS and Milli-Q water. The platelet adhesion was evaluated using SEM.

### Preparation of rats

Experiments on rats were conducted with the approval (KA2020-04) of the Animal Care Committee of the Korea Advanced Institute of Science and Technology. Specific pathogen–free Wistar rats (CrljOri:Wistar) were purchased from OrientBio. All rats were fed with ad libitum access to standard diet [Purina Milling Incorporated (PMI) laboratory diet] and water and were anesthetized by intraperitoneal injection of a combination of anesthetics [ketamine (80 mg/kg) and xylazine (12 mg/kg)] before all procedures were performed, and they were euthanized.

### PAD model

Before establishing the PAD model, we used a severe hind-leg ischemia model to validate the conformable microneedle pH meter. VICRYL 3-0 string was used for the tourniquet. After the conformable meter was applied to the appropriate region, the tourniquet was placed on the hip joint of the right hind leg and then fastened for 8 min. For development of the PAD model, the right femoral artery was exposed after anesthesia. The femoral artery was ligated with VICRYL 6-0, and then the muscle and skin were closed in the usual manner. One week later, right hind-leg claudication was observed. The pH was measured using a conformable microneedle pH meter on the affected leg. The data on the distribution of pH were calculated according to the sensitivity, which was measured using a separate PBS solution.

## Supplementary Materials

### This PDF file includes:

Figs. S1 to S14 References
