# Supplementary material for: Conformable microneedle pH sensors via the integration of two different siloxane polymers for mapping peripheral artery disease
Source: Sci Adv. 2021 Nov 26;7(48):eabi6290. doi: 10.1126/sciadv.abi6290 (PMC8626065; doi:10.1126/sciadv.abi6290)
Supplement: Supplementary file 1 — Figs. S1 to S14 References [file sciadv.abi6290_sm.pdf]

Science Advances
AAAS

Supplementary Materials for

Conformable microneedle pH sensors via the integration of two different siloxane polymers for mapping peripheral artery disease

Wonryung Lee*, Seung-hwan Jeong, Young-Woo Lim, Hyunhwan Lee, Joohyuk Kang, Hyunjae Lee, Injun Lee, Hyung-Seop Han, Shingo Kobayashi, Masaru Tanaka, Byeong-Soo Bae*

*Corresponding author. Email: wrlee@kist.re.kr (W.L.); bsbae@kaist.ac.kr (B.-S.B)

Published 26 November 2021, *Sci. Adv.* **7**, eabi6290 (2021)
DOI: 10.1126/sciadv.abi6290

This PDF file includes:

Figs. S1 to S14
References

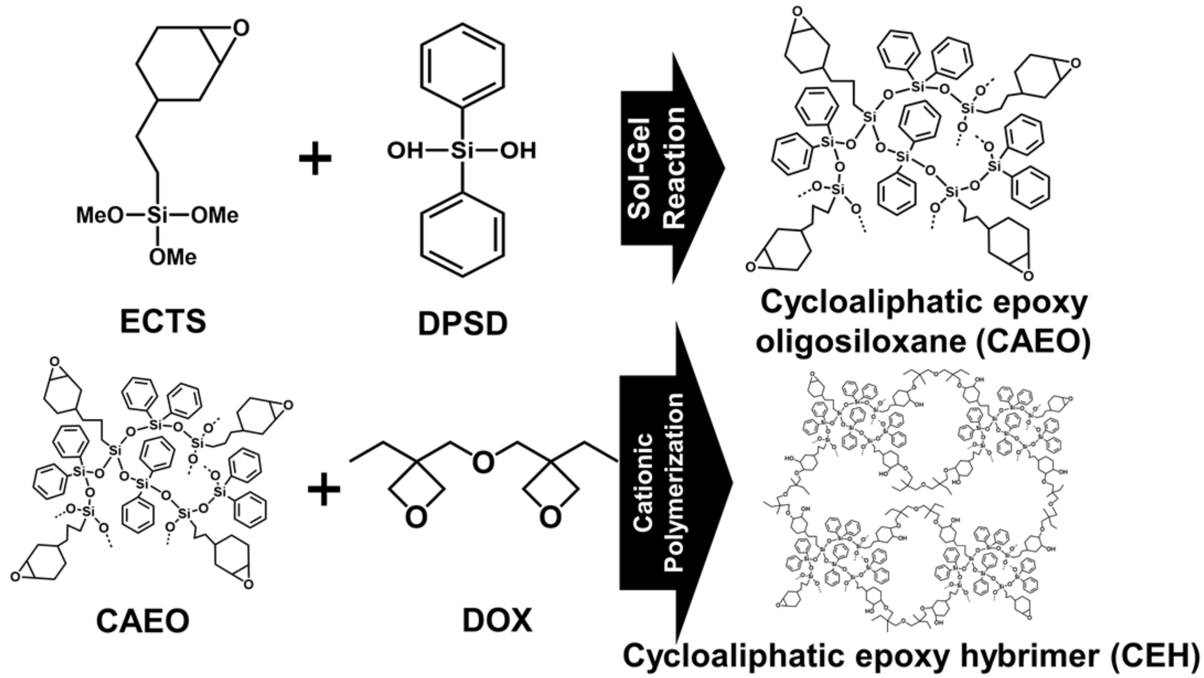

Fig. S1.

Process for synthesis of the epoxy siloxane polymer

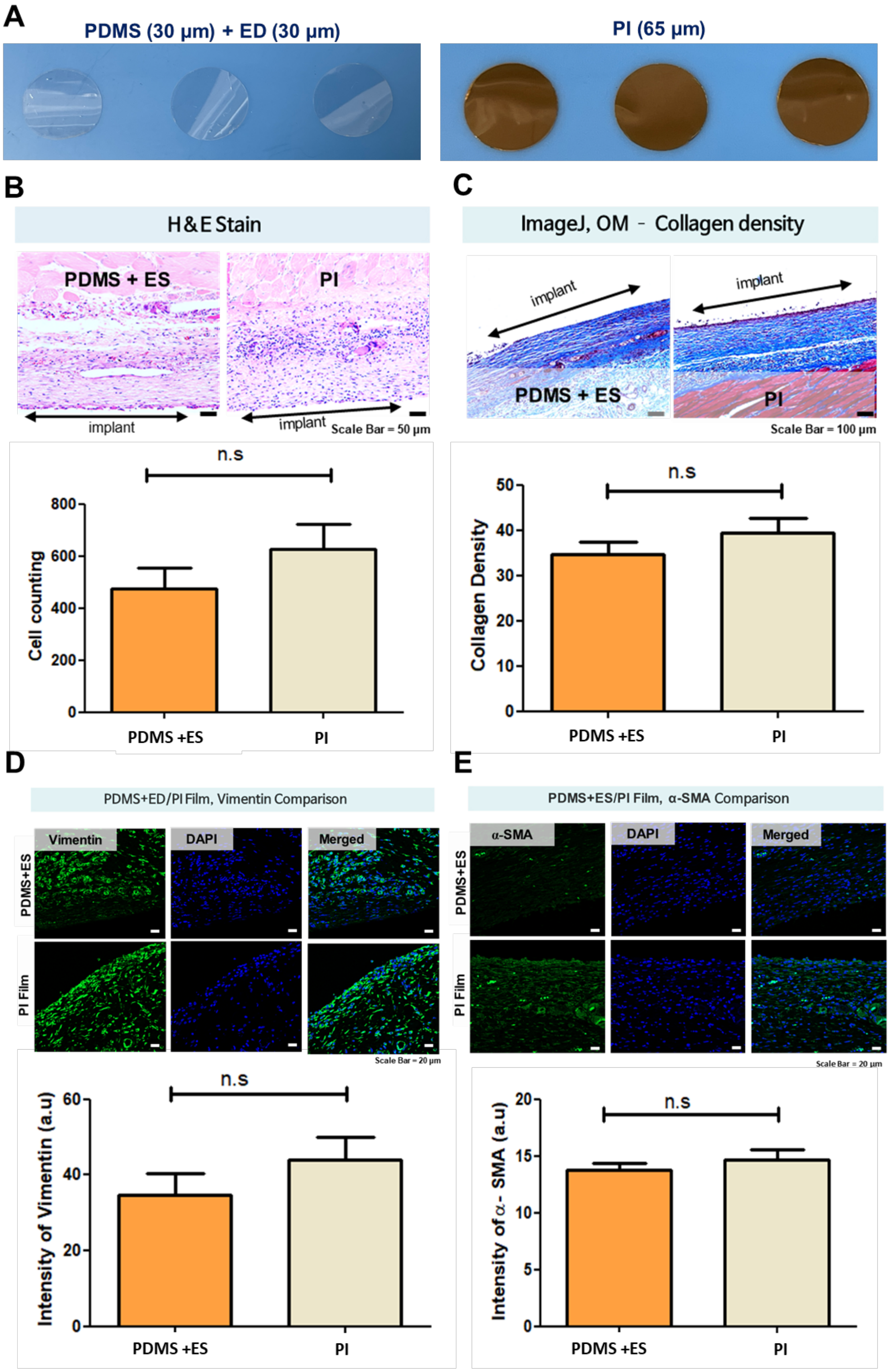

A
PDMS (30 µm) + ED (30 µm)
PI (65 µm)
B
H&E Stain
PDMS + ES
PI
implant
implant
Scale Bar = 50 µm
n.s
Cell counting
PDMS +ES
PI
C
ImageJ, OM - Collagen density
implant
implant
PDMS + ES
PI
Scale Bar = 100 µm
n.s
Collagen Density
PDMS +ES
PI
D
PDMS+ED/PI Film, Vimentin Comparison
Vimentin
DAPI
Merged
PDMS+ES
PI Film
Scale Bar = 20 µm
n.s
Intensity of Vimentin (a.u)
PDMS +ES
PI
E
PDMS+ES/PI Film, α-SMA Comparison
α-SMA
DAPI
Merged
PDMS+ES
PI Film
Scale Bar = 20 µm
n.s
Intensity of α- SMA (a.u)
PDMS +ES
PI

Fig. S2.

Various implant biocompatibility evaluation of PDMS + ES (EpoxySiloxane) and PI (Polyimide). The samples were implanted for 2 weeks.

(A) The sample of PDMS + ES (Epoxy Siloxane), diameter; 1cm. The PDMS was used as a stable support for ES film. (B) The sample of PI film, diameter; 1cm. (C) The biocompatibility evaluation using H & E staining. (D) The biocompatibility evaluation using measuring collagen density. (E) The biocompatibility evaluation using Vimentin staining. (F) The biocompatibility evaluation using α-SMA staining.

A series of in vivo experiments were performed to show the biocompatibility of the microneedle deposited with polyaniline. Following the approval from the institutional review board of Seoul National University Bundang Hospital, 10 Sprague Downey rats (8 weeks old) were injected with zoletil and received respiratory anesthesia before surgery. 3 cm incision was made on the back of the SD rats following the sterilization with betadine. Either PDMS+ED or PI samples with 2 cm in diameter were then inserted in the pocket made from the incision. The incision was sutured with 4-0 nylon. According to the in-house regulations, SD rats were administered to the post-surgical unit following the surgery and monitored for two weeks. SD rats were sacrificed after two weeks post-implantation and sample containing tissue was collected and fixed in 10% formalin for 24 hours. Microtome was used to create the 3 μm section slides for histological and immunohistological analysis. H&E staining of PDMS (Polydimethylsiloxane) +ED (Epoxy Siloxane) samples showed no significant difference in inflammation when compared to the PI samples. PI (Polyimide) samples were used as controls since countless studies show the excellent biocompatibility of the material. There were no sign of tissue inflammation and tissue necrosis for both samples. The density of the collagen also showed no significant difference between the two groups. Staining of Vimentin expression to quantify the fibroblast showed a slight increase for PDMS+ED samples but was not significantly different from the PI. Quantification of myofibroblast using the expression of α-SMA also showed similar trends, but there was no significant difference. In summary, a similar inflammatory score to the control material, increased collagen density, and increased fibroblast and myofibroblast provide evidence that confirmed the device's long-term safety.

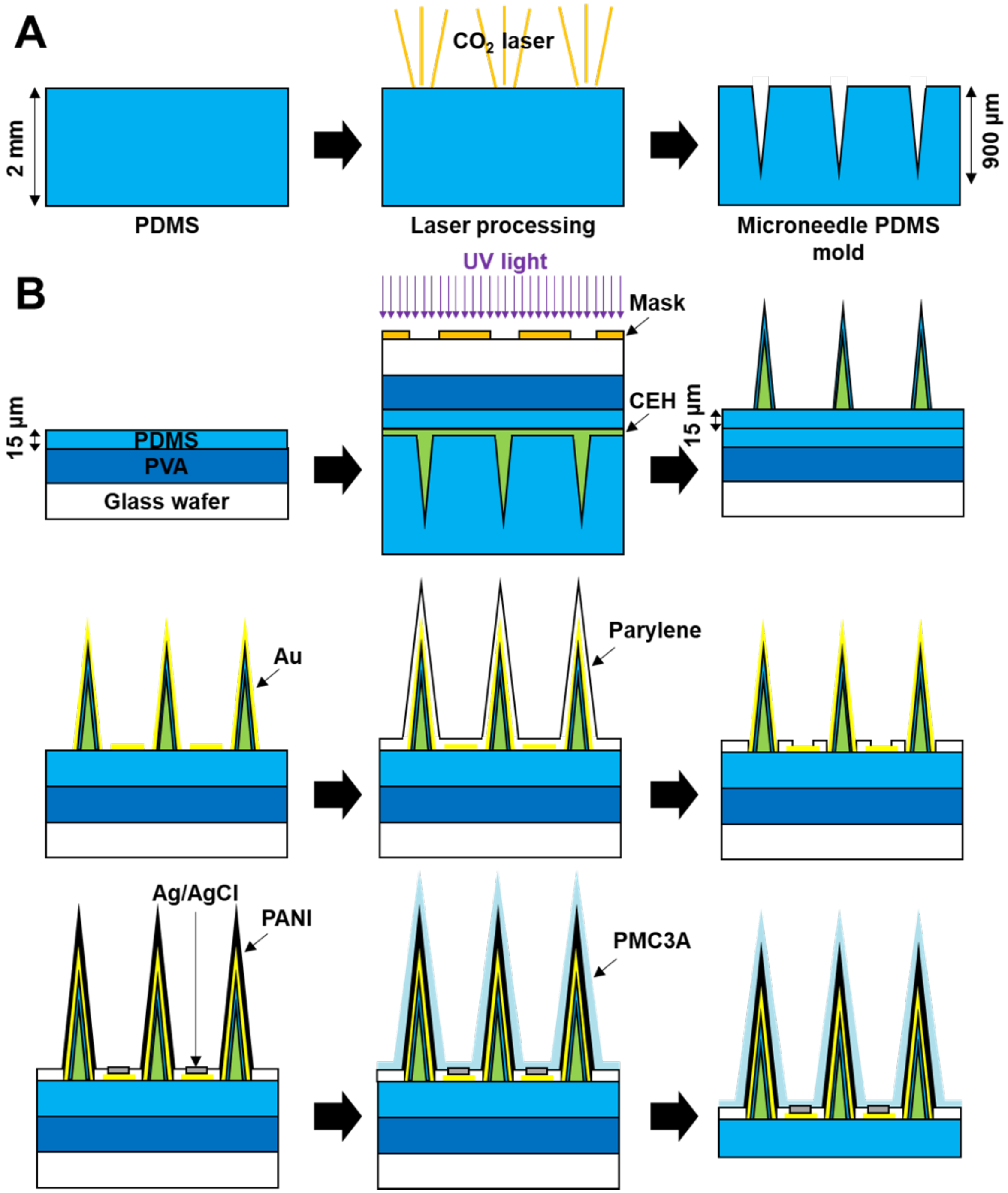

Fig. S3.

Schematic of fabrication of the flexible multi-microneedle pH-sensor array.

(A) Fabrication process for mold. (B) Fabrication process for integrated device.

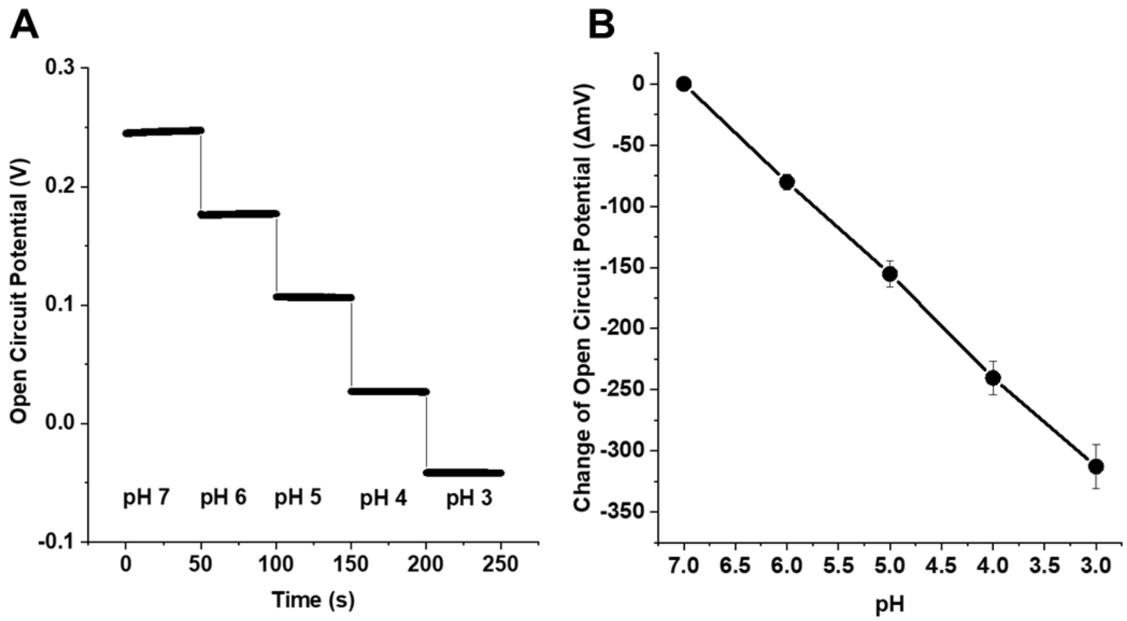

Fig. S4.

Electrical characteristics of pH sensor with microneedle in wide range of pH (pH 3~7).

(A) Open circuit potential (OCP)-time curve of single pH sensor with pH change. (B) Change of open circuit potential of 5 pH sensors.

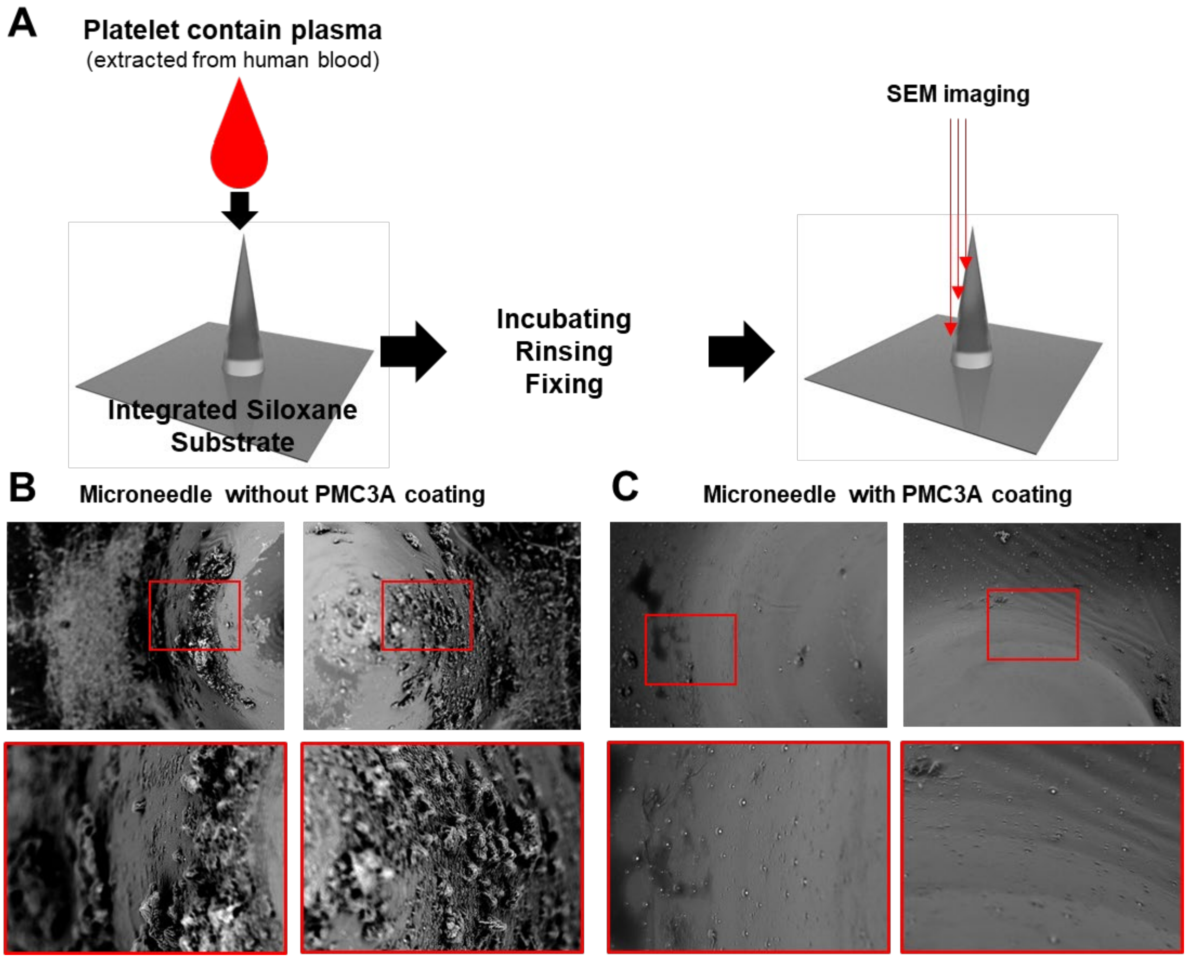

Fig. S5.

Blood-compatibility test on microneedle with PMC3A coating.

(A) Schematic of how to evaluate blood-compatibility of microneedles. (B) SEM image of a microneedle without PMC3A coating. (C) SEM image of a microneedle with PMC3A coating.

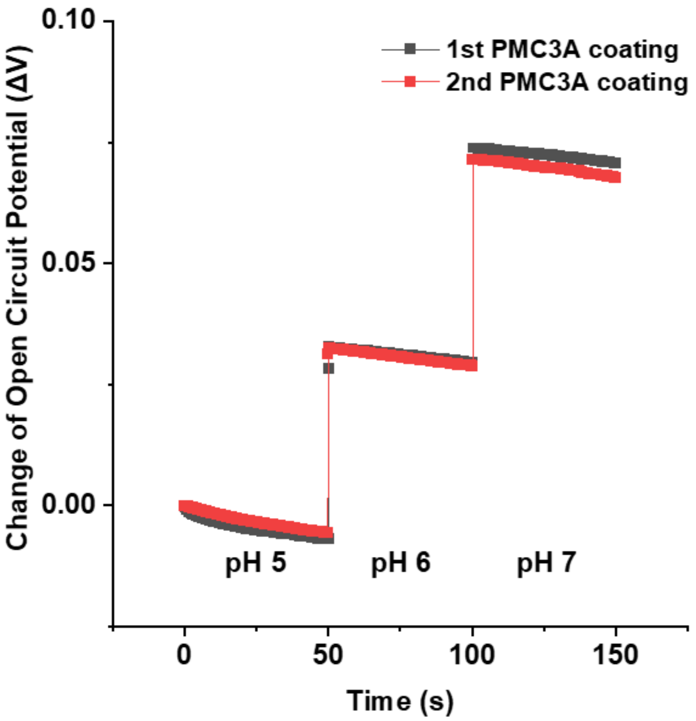

Fig. S6.

The comparison of sensitivity of pH sensors after re-coating of PMC3A. The pH sensors was cleaned to remove residues by ethanol, before re-coating.

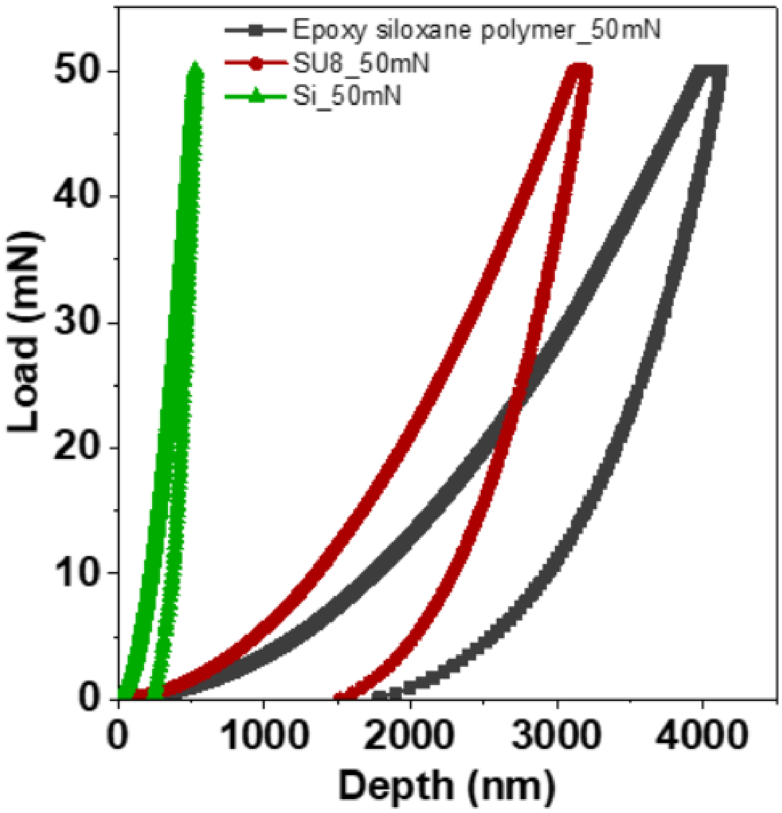

Fig. S7.

Comparison of representative load–displacement curves of nano-indentation characterization.

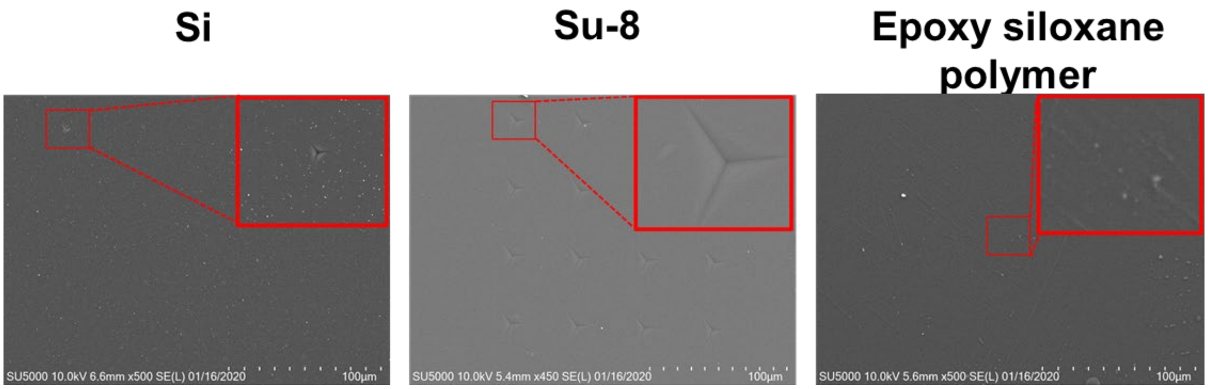

Fig. S8.
SEM images of residual impressions after the indentation tests.

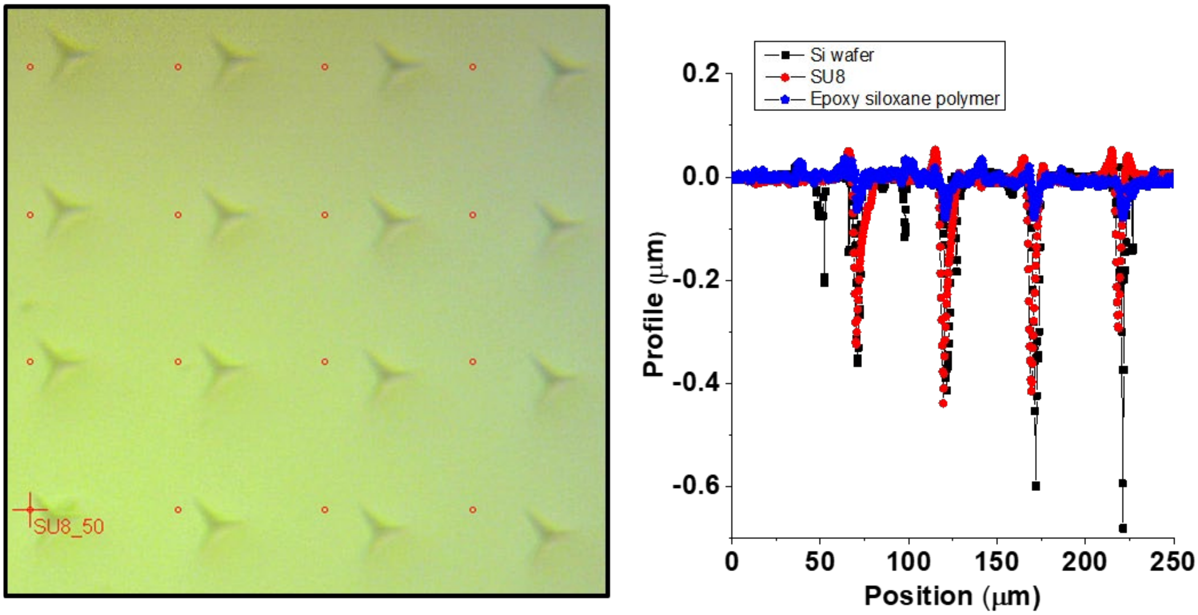

Fig. S9.

Microscope image and thickness profile of films after nanoindentation test. Thickness profiling was conducted by 3D image microscope (Keyence).

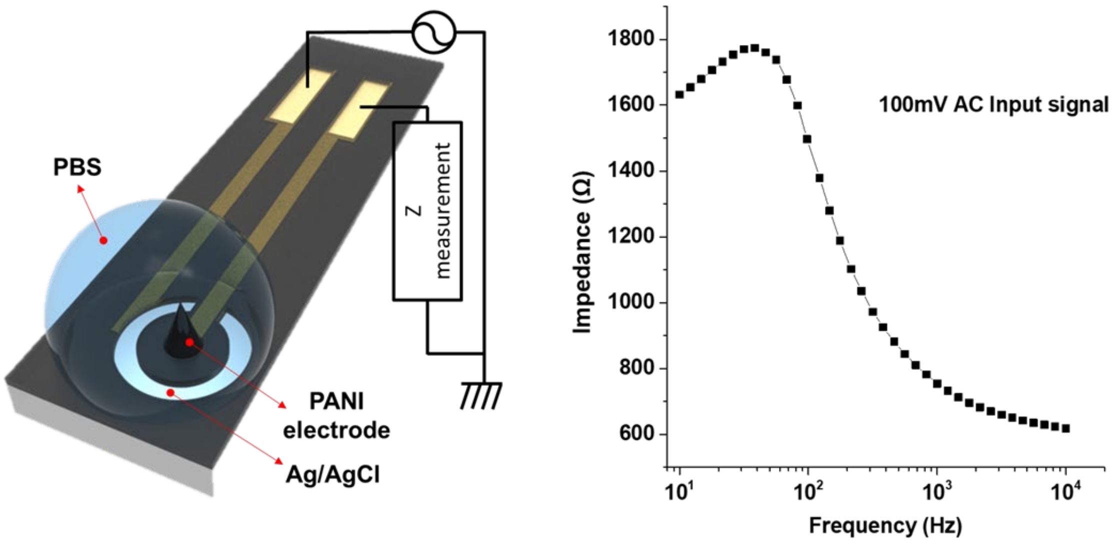

Fig. S10.

Impedance measurement for flexible microneedle PANI sensors with PMC3A coating.

(A) Schematic of measurement set-up. (B) Impedance-Frequency curves. A 100 mV amplitude input signal was applied.

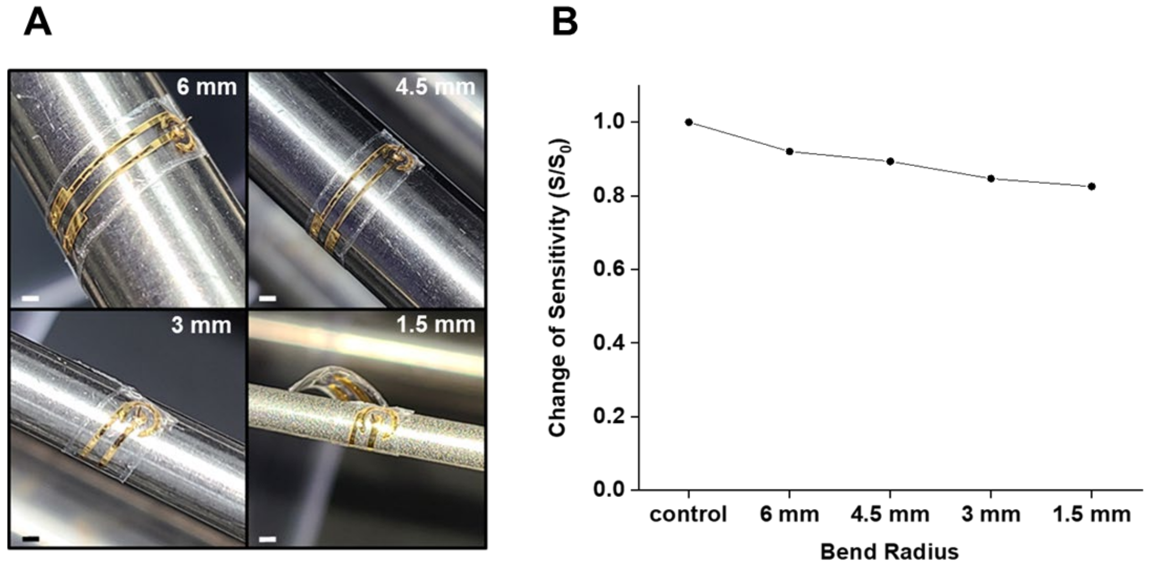

Fig. S11.

The mechanical stability evaluation of bending behavior of flexible microneedle pH sensors.

(A) Photograph image of flexible microneedle pH sensors for bending. Scale bar; 0.5 mm (B) Change of sensitivity of flexible microneedle pH sensors to bend radius.

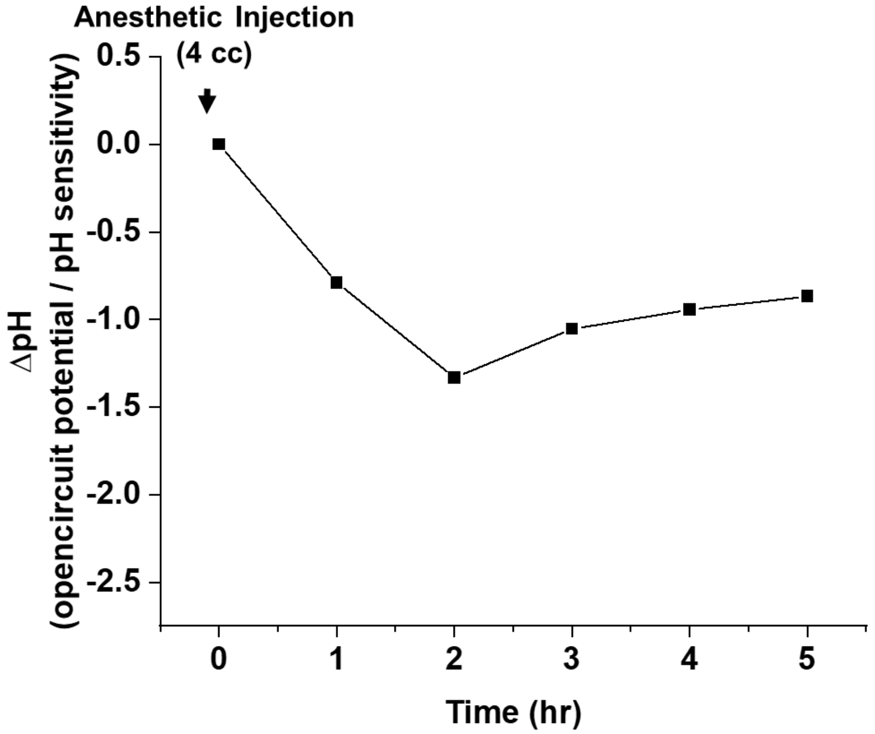

Fig. S12.

The long-term recording of change of pH on left leg of control rat model.

The conformable pH sensor was applied on thigh of anesthetized rat and allowed to measure the pH profile for five hours. Our device showed durable stability through the experiment. The rat was anesthetized with intraperitoneal injection of ketamine (35 mg/kg) and xylazine (65 mg/kg) as an induction dose and followed by administrations of maintenance doses of ketamine (7 mg/kg) and xylazine (13 mg/kg) with 1.5 hours of interval. Interestingly, pH showed gradually reduction during 2 hours after anesthesia, but recovered in some degree as time passed, which reflects respiratory acidosis due to respiratory suppression caused by relatively high induction dose of anesthetics and recovery to steady state with maintenance doses appealing minimal pH recovery. (*44*)

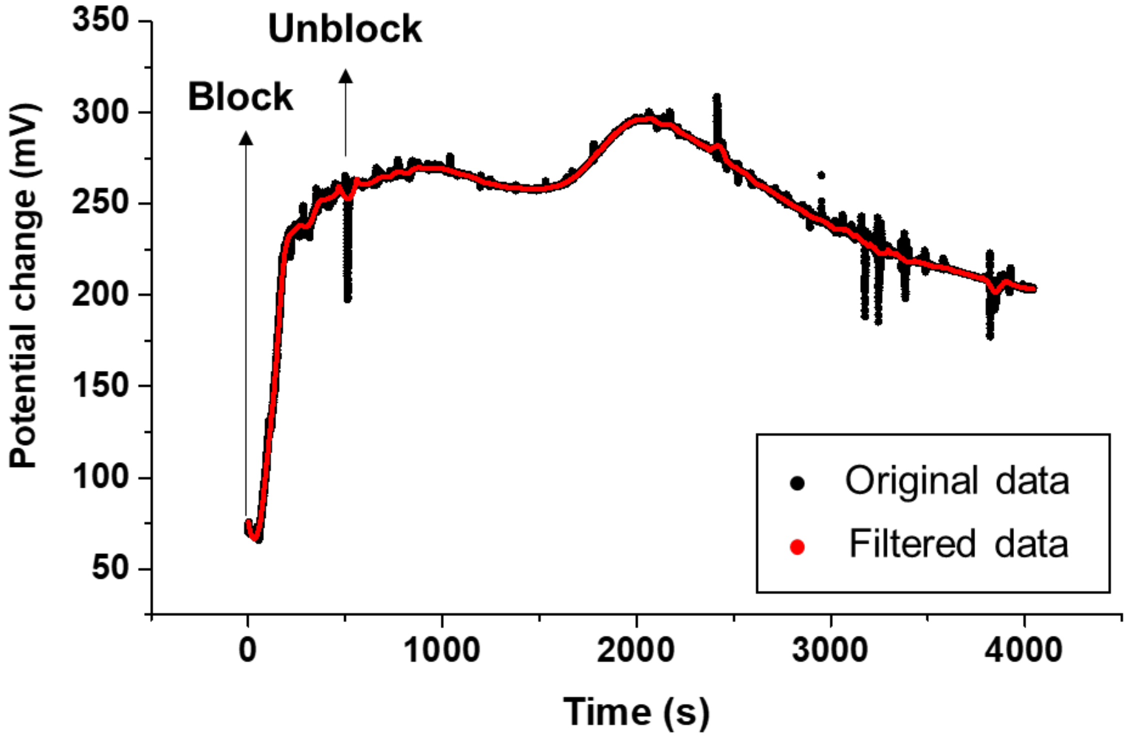

Fig. S13.

The potential change for peripheral vascular diseases model with ting artery for 4000 seconds, using microneedle PANI sensor. Filtering was conducted by Origin 9 using smooth tools. Savitzky-Golay method was used for this process. Points of window was 5, polynomial order was 2.

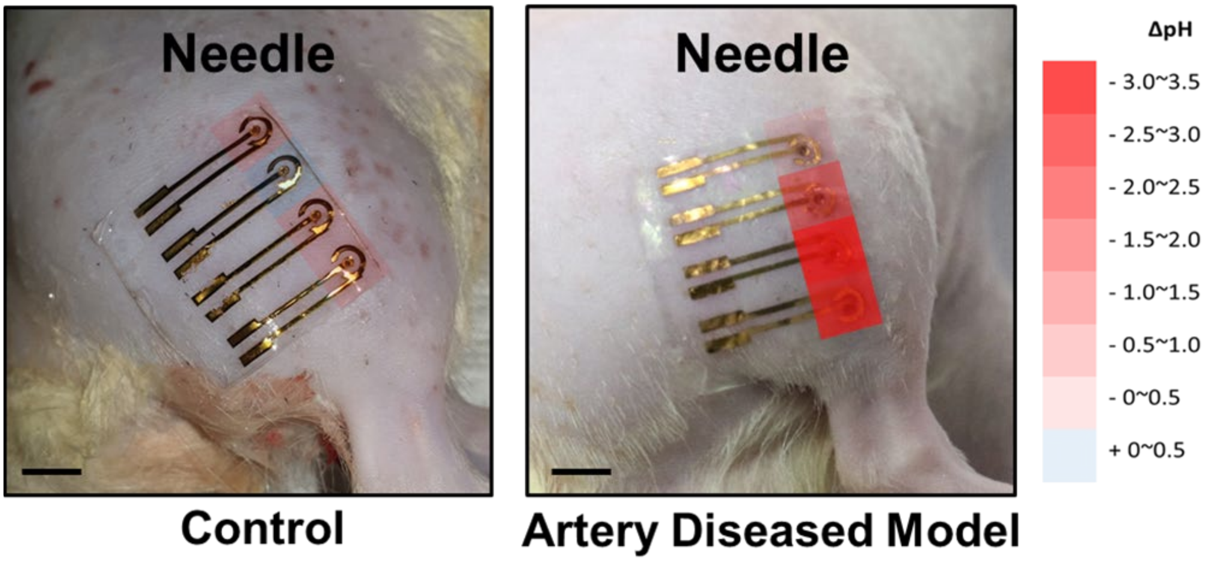

Fig. S14.

The comparison of pH distribution of control model and artery diseased model with microneedle sensors.

The conformable microneedle pH sensor showed gradual pH change of the lower limb from the thigh to the perineal area in artery diseased model, while control model exhibited constant pH distribution over measured area. “Photo Credit: Wonryung Lee, Korea Institute of Science and Technology.”

REFERENCES AND NOTES

1. W. R. Hiatt, F. G. R. Fowkes, H. Gretchen, J. S. Berger, I. Baumgartner, P. Held, B. G. Katona, K. W. Mahaffey, L. Norgern, W. S. Jones, J. Blomster, M. Millegard, C. Reist, M. R. Patel, Ticagrelor versus clopidogrel in peripheral artery disease. *N. Engl. J. Med.* **376**, 1487–1489 (2017).

2. F. G. R. Fowkes, D. Rudan, I. Rudan, V. Aboyans, J. O. Denenberg, M. M. McDermott, P. E. Norman, U. K. A. Sampson, L. J. Williams, G. A. Mensah, M. H. Criqui, Comparison of global estimates of prevalence and risk factors for peripheral artery disease in 2000 and 2010: A systematic review and analysis. *Lancet* **382**, 1329–1340 (2013).

3. F. G. R. Fowkes, V. Aboyans, F. J. I. Fowkes, M. M. McDermott, U. K. A. Sampson, M. H. Criqui, Peripheral artery disease: Epidemiology and global perspectives. *Nat. Rev. Cardiol.* **14**, 156–170 (2017).

4. G. Brevetti, G. Giugliano, L. Brevetti, W. R. Hiatt, Inflammation in peripheral artery disease. *Circulation* **122**, 1862–1875 (2010).

5. P. Z. Sun, J. S. Cheung, E. Wang, E. H. Lo, Association between pH-weighted endogenous amide proton chemical exchange saturation transfer MRI and tissue lactic acidosis during acute ischemic stroke. *J. Cereb. Blood Flow Metab.* **31**, 1743–1750 (2011).

6. E. Proksch, pH in nature, humans and skin. *J. Dermatol.* **45**, 1044–1052 (2018).

7. V. De Bergeyck, B. Naerhuyzen, A. M. Goffinet, C. Lambert de Rouvroit, A panel of monoclonal antibodies against reelin, the extracellular matrix protein defective in reeler mutant mice. *J. Neurosci. Methods* **82**, 17–24 (1998).

8. P. J. Rousche, R. A. Normann, Chronic recording capability of the utah intracortical electrode array in cat sensory cortex. *J. Neurosci. Methods* **82**, 1–15 (1998).

9. E. Forvi, M. Bedoni, R. Carabalona, M. Soncini, P. Mazzoleni, F. Rizzo, C. O'Mahony, C. Morasso, D. G. Cassarà, F. Gramatica, Preliminary technological assessment of microneedles-based dry electrodes for biopotential monitoring in clinical examinations. *Sensors Actuators A Phys.* **180**, 177–186 (2012).

10. C. Hegarty, A. McConville, R. J. McGlynn, D. Mariotti, J. Davis, Design of composite microneedle sensor systems for the measurement of transdermal pH. *Mater. Chem. Phys.* **227**, 340–346 (2019).

11. G. K. Mani, K. Miyakoda, A. Saito, Y. Yasoda, K. Kajiwara, M. Kimura, K. Tsuchiya, Microneedle pH sensor: Direct, label-free, real-time detection of cerebrospinal fluid and bladder pH. *ACS Appl. Mater. Interfaces* **9**, 21651–21659 (2017).

12. L. M. Strambini, A. Longo, S. Scarano, T. Prescimone, I. Palchetti, M. Minunni, D. Giannessi, G. Barillaro, Self-powered microneedle-based biosensors for pain-free high-accuracy measurement of glycaemia in interstitial fluid. *Biosens. Bioelectron.* **66**, 162–168 (2015).

13. K. B. Kim, W. C. Lee, C. H. Cho, D. S. Park, S. J. Cho, Y. B. Shim, Continuous glucose monitoring using a microneedle array sensor coupled with a wireless signal transmitter. *Sensors Actuators B Chem.* **281**, 14–21 (2019).

14. R. K. Mishra, K. Y. Goud, Z. Li, C. Moonla, M. A. Mohamed, F. Tehrani, H. Teymourian, J. Wang, Continuous opioid monitoring along with nerve agents on a wearable microneedle sensor array. *J. Am. Chem. Soc.* **142**, 5991–5995 (2020).

5. J. Gao, W. Huang, Z. Chen, C. Yi, L. Jiang, Simultaneous detection of glucose, uric acid and cholesterol using flexible microneedle electrode array-based biosensor and multi-channel portable electrochemical analyzer. *Sensors Actuators B Chem.* **287**, 102–110 (2019).

16. H. Lee, T. K. Choi, Y. B. Lee, H. R. Cho, R. Ghaffari, L. Wang, H. J. Choi, T. D. Chung, N. Lu, T. Hyeon, S. H. Choi, D. H. Kim, A graphene-based electrochemical device with thermoresponsive microneedles for diabetes monitoring and therapy. *Nat. Nanotechnol.* **11**, 566–572 (2016).

17. W. Li, R. N. Terry, J. Tang, M. R. Feng, S. P. Schwendeman, M. R. Prausnitz, Rapidly separable microneedle patch for the sustained release of a contraceptive. *Nat. Biomed. Eng.* **3**, 220–229 (2019).

18. W. Li, J. Tang, R. N. Terry, S. Li, A. Brunie, R. L. Callahan, R. K. Noel, C. A. Rodríguez, S. P. Schwendeman, M. R. Prausnitz, Long-acting reversible contraception by effervescent microneedle patch. *Sci. Adv.* **5**, eaaw8145 (2019).

19. M. A. Lopez-Ramirez, F. Soto, C. Wang, R. Rueda, S. Shukla, C. Silva-Lopez, D. Kupor, D. A. McBride, J. K. Pokorski, A. Nourhani, N. F. Steinmetz, N. J. Shah, J. Wang, Built-in active microneedle patch with enhanced autonomous drug delivery. *Adv. Mater.* **32**, e1905740 (2020).

20. D.-H. Kim, J. Viventi, J. J. Amsden, J. Xiao, L. Vigeland, Y. S. Kim, J. A. Blanco, B. Panilaitis, E. S. Frechette, D. Contreras, D. L. Kaplan, F. G. Omenetto, Y. Huang, K. C. Hwang, M. R. Zakin, B. Litt, J. A. Rogers, Dissolvable films of silk fibroin for ultrathin conformal bio-integrated electronics. *Nat. Mater.* **9**, 511–517 (2010).

21. M. Kaltenbrunner, T. Sekitani, J. Reeder, T. Yokota, K. Kuribara, T. Tokuhara, M. Drack, R. Schwödiauer, I. Graz, S. Bauer-Gogonea, S. Bauer, T. Someya, An ultra-lightweight design for imperceptible plastic electronics. *Nature* **499**, 458–463 (2013).

22. D. Wirthl, R. Pichler, M. Drack, G. Kettlguber, R. Moser, R. Gerstmayr, F. Hartmann, E. Bradt, R. Kaltseis, C. M. Siket, S. E. Schausberger, S. Hild, S. Bauer, M. Kaltenbrunner, Instant tough bonding of hydrogels for soft machines and electronics. *Sci. Adv.* **3**, e1700053 (2017).

23. M. Baumgartner, F. Hartmann, M. Drack, D. Preninger, D. Wirthl, R. Gerstmayr, L. Lehner, G. Mao, R. Pruckner, S. Demchyshyn, L. Reiter, M. Strobel, T. Stockinger, D. Schiller, S. Kimeswenger, F. Greibich, G. Buchberger, E. Bradt, S. Hild, S. Bauer, M. Kaltenbrunner, Resilient yet entirely degradable gelatin-based biogels for soft robots and electronics. *Nat. Mater.* **19**, 1102–1109 (2020).

24. T. Verho, C. Bower, P. Andrew, S. Franssila, O. Ikkala, R. H. A. Ras, Mechanically durable superhydrophobic surfaces. *Adv. Mater.* **23**, 673–678 (2011).

25. Q. Zhou, K. Lee, K. N. Kim, J. G. Park, J. Pan, J. Bae, J. M. Baik, T. Kim, High humidity- and contamination-resistant triboelectric nanogenerator with superhydrophobic interface. *Nano Energy* **57**, 903–910 (2019).

26. G. M. Choi, J. Jin, D. Shin, Y. H. Kim, J. H. Ko, H. G. Im, J. Jang, D. Jang, B. S. Bae, Flexible hard coating: Glass-like wear resistant, yet plastic-like compliant, transparent protective coating for foldable displays. *Adv. Mater.* **29**, 1700205 (2017).

27. G. Stavrinidis, K. Michelakis, V. Kontomitrou, G. Giannakakis, M. Sevrisarianos, G. Sevrisarianos, N. Chaniotakis, Y. Alifragis, G. Konstantinidis, SU-8 microneedles based dry electrodes for electroencephalogram. *Microelectron. Eng.* **159**, 114–120 (2016).

28. R. Wang, W. Zhao, W. Wang, Z. Li, A flexible microneedle electrode array with solid silicon needles. *J. Microelectromech. Syst.* **21**, 1084–1089 (2012).

29. R. Wang, X. Jiang, W. Wang, Z. Li, A microneedle electrode array on flexible substrate for long-term EEG monitoring. *Sensors Actuators B Chem.* **244**, 750–758 (2017).

30. Y. Shirosaki, K. Tsuru, S. Hayakawa, A. Osaka, M. A. Lopes, J. D. Santos, M. H. Fernandes, In vitro cytocompatibility of MG63 cells on chitosan-organosiloxane hybrid membranes. *Biomaterials* **26**, 485–493 (2005).

31. L. Ren, K. Tsuru, S. Hayakawa, A. Osaka, Novel approach to fabricate porous gelatin-siloxane hybrids for bone tissue engineering. *Biomaterials* **23**, 4765–4773 (2002).

32. J. R. Sempionatto, M. Lin, L. Yin, E. De, K. Pei, T. Sonsa-ard, A. N. D. L. Silva, A. A. Khorshed, F. Zhang, N. Tostado, S. Xu, J. Wang, An epidermal patch for the simultaneous monitoring of haemodynamic and metabolic biomarkers. *Nat. Biomed. Eng.* **5**, 737–748 (2021).

33. Y. Yang, Y. Song, X. Bo, J. Min, O. S. Pak, L. Zhu, M. Wang, J. Tu, A. Kogan, H. Zhang, T. K. Hsiai, Z. Li, W. Gao, A laser-engraved wearable sensor for sensitive detection of uric acid and tyrosine in sweat. *Nat. Biotechnol.* **38**, 217–224 (2020).

34. W. Gao, S. Emaminejad, H. Y. Y. Nyein, S. Challa, K. Chen, A. Peck, H. M. Fahad, H. Ota, H. Shiraki, D. Kiriya, D. H. Lien, G. A. Brooks, R. W. Davis, A. Javey, Fully integrated wearable sensor arrays for multiplexed in situ perspiration analysis. *Nature* **529**, 509–514 (2016).

35. Y. J. Hong, H. Lee, J. Kim, M. Lee, H. J. Choi, T. Hyeon, D. H. Kim, Multifunctional wearable system that integrates sweat-based sensing and vital-sign monitoring to estimate pre−/post-exercise glucose levels. *Adv. Funct. Mater.* **28**, 1805754 (2018).

36. Y. Mohd, R. Ibrahim, M. F. Zainal, in *2012 IEEE Symposium on Humanities, Science and Engineering Research* (2021), pp. 1301–1306.

37. M. Khalil, N. Liu, R. L. Lee, Super-Nernstian potentiometric pH sensor based on the electrodeposition of iridium oxide nanoparticles. *Int. J. Technol.* **9**, 446–454 (2018).

38. W. Lee, S. Kobayashi, M. Nagase, Y. Jimbo, I. Saito, Y. Inoue, T. Yambe, M. Sekino, G. G. Malliaras, T. Yokota, M. Tanaka, T. Someya, Nonthrombogenic, stretchable, active multielectrode array for electroanatomical mapping. *Sci. Adv.* **4**, eaau2426 (2018).

39. S. Diridollou, V. Vabre, M. Berson, L. Vaillant, D. Black, J. M. Lagarde, J. M. Grégoire, Y. Gall, F. Patat, Skin ageing: Changes of physical properties of human skin in vivo. *Int. J. Cosmet. Sci.* **23**, 353–362 (2001).

40. Y. Zhai, H. Petrowsky, J. C. Hong, R. W. Busuttil, J. W. Kupiec-Weglinski, Ischaemia-reperfusion injury in liver transplantation-from bench to bedside. *Nat. Rev. Gastroenterol. Hepatol.* **10**, 79–89 (2013).

41. M. T. Ghoneim, A. Nguyen, N. Dereje, J. Huang, G. C. Moore, P. J. Murzynowski, C. Dagdeviren, Recent progress in electrochemical pH-sensing materials and configurations for biomedical applications. *Chem. Rev.* **119**, 5248–5297 (2019).

42. P. Salvo, N. Calisi, B. Melai, V. Dini, C. Paoletti, T. Lomonaco, A. Pucci, F. Di Francesco, A. Piaggesi, M. Romanelli, Temperature- and pH-sensitive wearable materials for monitoring foot ulcers. *Int. J. Nanomedicine* **12**, 949–954 (2017).

43. S. Kobayashi, M. Wakui, Y. Iwata, M. Tanaka, Poly(ω-methoxyalkyl acrylate)s: Nonthrombogenic polymer family with tunable protein adsorption. *Biomacromolecules* **18**, 4214–4223 (2017).

44. T. M. Schwarzkopf, T. Horn, D. Lang, J. Klein, Blood gases and energy metabolites in mouse blood before and after cerebral ischemia: The effects of anesthetics. *Exp. Biol. Med.* **238**, 84–89 (2013).
